# Case-control study on risk factors for acute hepatitis E in Germany, 2012 to 2014

**DOI:** 10.2807/1560-7917.ES.2018.23.19.17-00469

**Published:** 2018-05-10

**Authors:** Mirko Faber, Mona Askar, Klaus Stark

**Affiliations:** 1 Department for Infectious Disease Epidemiology, Robert Koch Institute, Berlin, Germany

**Keywords:** Hepatitis E, Germany, human, case-control study, risk factors

## Abstract

Notified cases of hepatitis E have increased 40-fold in the past 10 years in Germany. Food safety is a major concern as hepatitis E virus (HEV) RNA has been detected in ready-to-eat retail-level food products. The objective of this case–control study was to assess risk factors for autochthonous symptomatic hepatitis E and explore reasons for delays in diagnosis. **Methods:** Demographic, clinical and exposure data from notified hepatitis E cases and individually matched population controls were collected in semi-standardised telephone interviews. Conditional logistic regression analysis was used to calculate matched odds ratios (mOR) and population attributable fractions (PAF). **Results:** In total, 270 cases and 1,159 controls were included (mean age 53 years, 61% men in both groups). Associated with disease were: consumption of undercooked pork liver, pork, wild boar meat, frankfurters, liver sausage and raw vegetables; contact with waste water (occupational) and various host factors (mORs between 1.9 and 34.1, p value < 0.03). PAF for frankfurters and liver sausage were 17.6%, and 23.6%, respectively. There were statistically significant differences in the clinical presentation and hospitalisation proportion of acute hepatitis E in men and women. Diagnosis was preceded by more invasive procedures in 29.2% of patients, suggesting that hepatitis E was not immediately considered as a common differential diagnosis. **Conclusions:** Our study suggests that there are indeed sex-specific differences in disease development and lends important epidemiological evidence to specific ready-to-eat pork products as a major source for autochthonous hepatitis E. A review of existing consumer recommendations and production methods may be indicated.

## Introduction

Hepatitis E in western and central Europe is predominantly a zoonosis caused by infection with genotype 3 hepatitis E virus (HEV). Domestic pigs and wild boars have been identified as the main reservoir animals. Consumption of raw or undercooked meat, offal and meat products have been implicated as risk factors for hepatitis E in humans [1-6]. However, several other routes of transmission have been demonstrated or suggested: e.g. consumption of shellfish, receiving blood products or transplants from an infected donor, contact with infected animals or contaminated water [7-11]. Infection is most often asymptomatic, with clinical hepatitis more commonly reported among men over 50 years of age or persons with pre-existing liver disease, suggesting that host factors play an important role [12].

The relative importance of the various transmission routes and host factors remains unknown. Studies involving larger samples of clinical cases and adequate control persons are lacking and results on alimentary risk factors in particular are not easily transferrable due to differences in alimentary habits between countries.

In Germany, hepatitis E is a notifiable disease according to the Protection against Infection Act of 2001 [13], and the number of notified hepatitis E cases (symptomatic patient with laboratory confirmation) has increased 40-fold in the past 10 years. Of the 2,943 cases notified in 2017, 57% were in men, 75% in the age group 40–79 years and 93% in persons with no travel history [14]. Seroprevalence and seroincidence were estimated at 16.8% and 3.9 infections per 1,000 population per year, respectively, indicating that HEV is highly endemic in Germany [15]. The increase in notified cases in recent years is likely attributable to an increasing awareness of the disease, indicated by a stable or slightly decreasing seroprevalence in the German adult population [16]. Thus, the unbroken trend towards higher annual case numbers suggests that hepatitis E is still underdiagnosed. Food safety is a concern as cooking temperatures of 71 °C for 20 min are required to fully inactivate the virus [17] and HEV RNA has been detected in ready-to-eat food products, including raw sausage and liver sausage at retail level [18].

Our objectives were to identify risk factors for autochthonous hepatitis E infections in Germany, and to discover reasons for delays in diagnosing hepatitis E in order to develop recommendations for further prevention.

## Methods

### Study design

We conducted a country-wide case-control study among hepatitis E cases aged 18 years or older notified between January 2012 and January 2014 and matching controls. All subjects were enrolled after obtaining informed consent. The study was approved by the ethics committee of the Charité university hospital, Berlin and the Federal Commissioner for Data Protection and Freedom of Information.

Hepatitis E patients were recruited by local health departments during their routine case investigations. If informed consent was provided, the patient's contact data was faxed to the Robert Koch Institute (RKI) so that a telephone interview could be conducted. A case was defined as a person notified to the local health department during the study period with a laboratory-confirmed HEV-infection (IgM or PCR positive) presenting with at least one of the following symptoms: fever, jaundice or abdominal pain. Case-patients were excluded from data analysis if the date of symptom onset was unknown or if within 2 months before onset of symptoms they had travelled within Europe for more than 14 days or for any duration outside Europe.

We aimed to match each case-patient with four control participants of the same sex, age group (18–34, 35–49, 50–69 and ≥ 70 years) and post code. The control participants were recruited using a database of publicly available telephone numbers (mobiles and landlines) with known post code. Interviewers asked to speak to a respondent within the household matching the respective case criteria. If no household member with the sought-after criteria was available the call was ended and additional telephone numbers were dialled until quota requirements were met.

#### Data collection

Cases and controls were interviewed by trained study personnel via telephone using a standardised questionnaire. The questionnaire was created after a literature review and inquired about known risk factors for hepatitis E with a focus on alimentary exposures (e.g. meat and meat products or following a specific diet) but also included environmental exposures (contact with animals or sewage), person-to-person contact and host factors such as pre-existing conditions, alcohol consumption and prescription and over-the-counter medicines, course of disease, diagnostics and symptoms.

The telephone interview of the control persons resembled the interview for case-patients (except diagnostics, disease course and symptoms). Questions about possible exposures referred to the 2 months before disease onset (case-patients) or before interview (controls).

#### Data analysis

Hypotheses regarding differences between groups were tested using chi-squared or Wilcoxon rank-sum test. To investigate risk factors for autochthonous hepatitis E, univariable analysis including a total of 114 variables was conducted by computing matched odds ratios (mOR) and 95% confidence intervals (CIs) using the Mantel-Haenszel method implemented in STATA's mhodds command. We calculated the number of days between the date of onset, the date of the first visit to a physician, the date of blood sampling and the date of diagnosis as reported by the case in order to discover reasons for delayed diagnoses.

Variables with an adjusted odds ratio (aOR) > 1 and a p value < 0.2 were selected for multivariable logistic regression modelling. In a first step, multivariable submodels were developed for pre-existing conditions, animal contact, consumption of meat, meat products (including sausages), other food items, special diets, animal contact, the patient’s housing environment and environmental exposures. A manual backward selection procedure was used with a cut-off of p < 0.05. Thereafter, the submodels were combined in one final model using the described backward selection procedure. Finally, previously eliminated variables were reintroduced in a stepwise fashion and kept if significantly associated with disease (p < 0.05). The variable age was always forced into the respective models.

The population attributable fraction (PAF) denotes the proportion of cases that could be prevented by removing the respective risk factor, assuming it is causal and its effect was measured accurately. The PAF and the respective 95% confidence intervals were computed for all risk factors/variables of the multivariable model using STATA's punafcc command [19].

## Results

### Study population

During the study period, 370 cases were interviewed, corresponding to 43.8% of all 845 cases of hepatitis E notified to RKI by local health authorities. Of these, 270 (73.0%) were included in the data analysis, while 100 (27.0%) were excluded because of travel history, missing symptoms and/or unknown date of disease onset. Case-patients included in the data analysis did not significantly differ from the overall group of cases notified through the German surveillance system (n = 845) or their individually matched controls (n = 1,159) in terms of age group, sex or region of living (Table 1).

**Table 1:** Age group, sex and regional distribution of hepatitis E cases notified through the Germany surveillance system, cases included in the risk factor analysis and individually matched controls, Germany, January 2012–January 2014

	Notified cases (n=845)	%	Cases in CCS (n=270)	%	Controls (n=1,159)	%
Age group (years)						
18–34	129	15.3	28	10.4	138	11.9
35–49	243	28.8	79	29.3	312	26.9
50–64	305	36.1	112	41.5	435	37.5
≥65	168	19.9	51	18.9	274	23.6
Sex						
Female	313	37.0	105	38.9	451	38.9
Male	532	63.0	165	61.1	708	61.1
Region^a^						
North	177	20.9	42	15.6	172	14.8
West	267	31.6	83	30.7	375	32.4
East	248	29.3	88	32.6	375	32.4
South	153	18.1	57	21.1	237	20.4

CCS: case-control study.

^a^ Named regions included the following German states: North – Bremen, Hamburg, Lower Saxony, Mecklenburg-Western Pomerania, Schleswig-Holstein; West – Hesse, North Rhine-Westphalia, Rhineland-Palatinate, Saarland; East – Brandenburg, Berlin, Saxony, Saxony-Anhalt, Thuringia ; South – Baden-Württemberg, Bavaria.

The proportion of hospitalised patients was 73.7% (199/270) and differed significantly between women (63.8%, 67/105) and men (80.0%, 132/165), p < 0.01. Sex differences were also evident regarding the frequency of symptoms reported by participants. Particularly, symptoms related to the accumulation of bilirubin, e.g. jaundice, were more frequent among men, while gastrointestinal symptoms were more prevalent among women (Table 2). The median duration of illness was 34 days and the median number of work days missed by persons who reported missing at least 1 day of work (121/270, 44.8%) was 20 days. Both are minimum estimates, because for patients who still had symptoms at the time of the interview (168/258, 65.1%), only the period between disease onset and interview was counted.

**Table 2:** Proportion of hospitalisation and frequency of symptoms among autochthonous and symptomatic cases of hepatitis E by sex, Germany, 2012–2014 (n = 270)

	All cases (n = 270)	%	Female (n = 105)	%	Male (n = 165)	%	Chi-squared p value
**Hospitalised**	199/270	**73.7**	67/105	63.8	132/165	80.0	p < 0.01
**Fever > 38 °C**	84/265	**31.7**	34/103	33.0	50/162	30.9	NS
**Abdominal pain**	97/269	**36.1**	48/105	45.7	49/164	29.9	p < 0.01
**Nausea**	129/269	**48.0**	60/105	57.1	69/164	42.1	p < 0.05
**Vomiting**	65/267	**24.3**	30/102	29.4	35/165	21.2	NS
**Diarrhoea**	71/268	**26.5**	30/104	28.9	41/164	25.0	NS
**Loss of appetite**	177/269	**65.8**	77/105	73.3	100/164	61.0	p < 0.05
**Headache**	102/268	**38.1**	47/104	45.2	55/164	33.5	NS
**Fatigue**	218/270	**80.7**	84/105	80.0	134/165	81.2	NS
**Upper or lower extremity pain**	108/265	**40.8**	44/104	42.3	64/161	39.8	NS
**Generalised pruritus**	117/267	**43.8**	44/104	42.3	73/163	44.8	NS
**Jaundice (eyes)**	145/269	**53.9**	42/105	40.0	103/164	62.8	p < 0.01
**Jaundice (skin)**	128/266	**48.1**	36/102	35.3	92/164	56.1	p < 0.01
**Dark urine**	206/267	**77.2**	66/105	62.9	140/162	86.4	p < 0.01
**Clay-coloured stool**	143/263	**54.4**	48/103	46.6	95/160	59.4	p < 0.05

NS: no statistically significant association.

### Risk factors associated with autochthonous hepatitis E

In the univariable analysis, 39 of 114 variables were positively associated (p < 0.2; OR > 1) with illness including consumption of various meat products such as wild boar and pork products that had not been fully cooked when consumed, several kinds of sausages and raw vegetables but also occupational contact with waste water and various pre-existing conditions of the liver (Table 3). Other meat products and fully cooked items appeared to be generally protective (OR < 1), and the remaining items were not associated with disease (data not shown).

**Table 3:** Risk factors for symptomatic hepatitis E virus infection in persons without travel history (univariable and multivariable logistic regression analysis), Germany, January 2012–2014 (n = 1,429)

Risk factor	Cases (n = 270)	%	Controls (n = 1,159)	%	Univariable mOR	95% CI	Multivariable mOR	95% CI	PAF	95% CI
Consumption of meat										
Minced pork^a^	42/269	15.6	130/1,157	11.2	1.6	1.1–2.4	NA			
Minced pork and beef (mix) ^a^	56/266	21.1	197/1,152	17.1	1.4	0.97–1.94	NA			
Pork (e.g. roast)	143/268	53.4	529/1,159	45.6	1.4	1.03–1.81	NA			
Pork (e.g. roast)^a^	16/263	6.1	21/1,158	1.8	3.9	1.9–7.9	3.0	1.4–6.5	0.04	0.01–0.07
Wild boar^a^	8/265	3.0	10/1,135	0.9	3.3	1.2–8.6	3.7	1.1–12.4	0.02	0–0.04
Rabbit^a^	2/265	0.8	2/1,135	0.2	3.6	0.5–23.5	NA			
Pork liver^a^	12/263	4.6	14/1,135	1.2	4.3	1.9–10.1	5.3	1.8–15.7	0.04	0.01–0.07
Pork offal (excluding liver)^a^	2/259	0.8	1/1,135	0.1	11.2	0.7–171.2	NA			
Beef liver^a^	6/265	2.3	11/1,134	1.0	2.6	0.9–7.7	NA			
Beef offal (excluding liver)^a^	2/264	0.8	1/1,135	0.1	8	0.7–88.2	NA			
Home-butchered meat	15/268	5.6	40/1,153	3.5	1.8	0.96–3.20	NA			
Consumption of meat products										
Spreadable sausages made of raw meat (e.g. Teewurst, Mettwurst)	94/268	35.1	307/1,158	26.5	1.5	1.1–2.0	NA			
Raw ham	165/270	61.1	600/1,158	51.8	1.4	1.1–1.0	NA			
Cooked cured products (eg., cooked ham)	109/269	40.5	349/1,159	30.1	1.6	1.2–2.1	NA			
Boiled sausage	141/269	52.4	496/1,158	42.8	1.5	1.1–2.0	NA			
Sülzwurst (sausage containing meat jelly)	24/269	8.9	73/1,157	6.3	1.6	0.95–2.59	NA			
Liver sausage or liver pâté	114/270	42.2	324/1,159	28.0	1.9	1.5–2.6	2.1	1.5–3	0.24	0.12–0.33
Boiled sausage (e.g. frankfurter, wiener)	97/267	36.3	282/1,159	24.3	2.0	1.4–2.7	1.9	1.3–2.7	0.18	0.08–0.26
Consumption of other food items										
Raw vegetables	222/269	82.5	805/1,157	69.6	2.0	1.4–2.8	1.9	1.3–2.9	0.39	0.17–0.56
Animal contact										
Dogs	58/267	21.7	204/1,159	17.6	1.3	0.9–1.8	NA			
Cats	65/265	24.5	221/1,159	19.1	1.4	1.01–1.89	NA			
Domestic pigs	4/265	1.5	8/1,159	0.7	2.2	0.7–7.5	NA			
Cattle	6/266	2.3	8/1,159	0.7	3.5	1.8–10.5	NA			
Environmental exposure										
Contact with waste water (non-occupational)	4/264	1.5	28/1,158	2.4	0.7	0.2–2.0	NA			
Contact with waste water (occupational)	20/267	7.5	18/1,159	1.6	5.2	2.7–10.1	5.5	2.1–13.9	0.06	0.02–0.1
Pre-existing conditions										
Diabetes mellitus	46/268	17.2	99/1,157	8.6	2.3	1.5–3.4	3.3	1.8–5.8	0.11	0.06–0.17
Autoimmune diseases	27/261	10.3	68/1,141	6.0	1.9	1.2–3.1	NA			
Malignant diseases	22/267	8.2	66/1,154	5.7	1.4	0.8–2.4	NA			
Gastrointestinal disease (not ulcerative colitis or Crohn's disease)	46/267	17.2	77/1,154	6.7	2.9	1.9–4.3	2.4	1.4–4.2	0.11	0.04–0.17
Congenital diseassses	8/268	3.0	15/1,152	1.3	2.3	0.99–5.55	NA			
Allergies	100/266	37.6	355/1,153	30.8	1.4	1.01–1.80	NA			
Acid-reducing drugs	60/268	22.4	185/1,155	16.0	1.5	1.1–2.1	NA			
Hepatitis B	5/265	1.9	3/1,155	0.3	10.0	1.9–51.8	29.2	4.9–175.3	0.02	0–0.03
Chronic liver infections (non-HBV/HCV)	7/265	2.6	6/1,155	0.5	5.4	1.6–18.7	NA			
Chronically elevated liver enzymes	24/270	8.9	30/1,151	2.6	4.4	2.3–8.3	6.1	2.9–12.6	0.07	0.04–0.11
Fatty liver disease (non-alcoholic)	16/270	5.9	34/1,151	3.0	1.9	1.04–3.47	NA			
Fatty liver disease (alcoholic)	7/270	2.6	5/1,151	0.4	9.1	2.2–36.8	NA			
Liver cirrhosis	9/270	3.3	2/1,151	0.2	36.0	4.5–285.4	34.1	3.1–372.2	0.03	0.01–0.06
Liver diseases, other	10/270	3.7	25/1,151	2.2	1.9	0.9–4.3	NA			

CI: confidence interval; HBV: hepatitis B virus; HCV: hepatitis C virus; mOR: matched odds ratio; PAF: population attributable fraction; NA: not applicable (variable is not part of the final multivariable model).

^a^ Consumed raw, rare or otherwise not fully cooked.

Only variables with an adjusted odds ratio (aOR) > 1 and a p value < 0.2 in the univariable analysis are shown.

In the multivariable model, variables related to exposure as well as host factors were significantly associated with disease. Among the exposures were pork (mOR: 3.0, 95% CI: 1.4–6.6) and wild boar meat (mOR: 3.7, 95% CI: 1.1–12.4) that was not fully cooked at the time of consumption, but also sausages that are intended to be eaten without further preparation (ready-to-eat), such as liver sausage or liver pâté (mOR: 2.1; 95%CI: 1.5–3.0) and frankfurter/wiener types (mOR: 1.9; 95%CI: 1.3–2.7). Additional exposures associated with disease were consumption of raw vegetables (mOR: 1.9, 95%CI: 1.3–2.9) and occupational contact with waste water (mOR: 5.5, 95% CI: 2.1–13.9). Among host factors were various pre-existing conditions of the liver, diabetes mellitus and gastrointestinal diseases other than Crohn's disease or ulcerative colitis (Table 3). The highest PAFs were found for liver pâté or liver sausage, boiled sausages (i.e. frankfurter or wiener) and raw vegetables.

Among cases, 1 of 266 (0.4%) followed a diet avoiding the consumption of pork or pork products (vegetarian, vegan, halal or kosher), compared with 67 of 1,155 controls (5.8%). In a single risk analysis, dietary exposure to pork was significantly associated with hepatitis E (mOR = 17.4; 95% CI: 2.4–127.9; p < 0.01). The PAF was 93.9% (95% CI: 0.56–0.99). Cases and controls did not differ in following any other special diet (3.5% cases vs 3.4% controls).

### From disease onset to diagnosis

Among 239 of 261 (91.6%) cases with available information, a positive serological test result was obtained and 42 (16.1%) were positive by PCR. From symptom onset to diagnosis of hepatitis E as reported by cases, it took a median of 19 days (interquartile range (IQR): 12–29 days). Cases reported first visit to a physician after a median of 5 days of symptoms (IQR: 2–12). In the majority of cases (218/254, 85.8%), physicians took blood samples at this first visit or the day thereafter. Median number of days between visit and blood sampling for all patients was 0 days (IQR: 0–0), but it took another 9 days (median, IQR: 5–16 days) until the diagnosis of hepatitis E was made. Eighty-five per cent of cases (222/260) reported seeing two or more practitioners before receiving a diagnosis of hepatitis E, 23.8% (62/260) saw three or more. Of all cases included in the data analysis, 168 (62%) reported receiving a preliminary diagnosis from their physician before the diagnosis of hepatitis E was made, including: hepatitis of unknown origin (28.6%, n = 48), viral hepatitis (17.9%, n = 30), cholecystitis (25.0%, n = 42), drug-induced hepatitis (10.7%, n = 18), gastrointestinal disease (8.9%, n = 15) and autoimmune hepatitis (7.1%, n = 12). Before the diagnosis was made serologically or by PCR, cases reported receiving the following investigations: ultrasound (91.5%, n = 246), stool (11.5%, n = 31) or urine diagnostics (17.4%, n = 46), CT (11.5%, n = 31), MRT (8.9%, n = 24), conventional X-ray (9.3%, n = 25), gastro- or colonoscopy (19.6%, n = 53), biopsy of the liver (17.4%, n = 47).

## Discussion

To our knowledge, this is the largest population-based study on risk factors for acute hepatitis E in Europe, involving 270 cases representative of all notified hepatitis E cases in the study period and over 1,100 closely matched controls. All cases included in our study had acute, symptomatic hepatitis E, setting it apart from serological studies. It has been suggested the inoculum required to cause symptomatic HEV infection is 1,000-fold higher than that leading to asymptomatic seroconversion. Thus, exposures relevant for symptomatic and asymptomatic infection may differ enough to be considered separately [20,21].

We found that risk factors for autochthonous hepatitis E in Germany include but are not limited to the consumption of undercooked pork liver, pork and wild boar meat. The consumption of two popular, ready-to-eat pork products, liver sausage or pâté and frankfurter/wiener type sausage, were significantly associated with acute hepatitis E and, in our final regression model, 23.6% and 17.6% of acute hepatitis E was attributable to these two vehicles, respectively.

Liver sausage and frankfurter/wiener type sausages available in Germany are cooked or parboiled during production and are generally assumed to be free of intact virions able to cause infection [18]. These products have thus to be distinguished from products that are expected to be cooked by the consumer before consumption, such as the figatellu raw-liver sausage implicated in several outbreaks in France [2,3] or the sausages from a major supermarket chain implicated in a case-control-study in the United Kingdom [4].

Previous studies have detected HEV genome (RNA) in various commercial pork products such as in 22% of liver sausages bought in supermarkets in Berlin, 47% of pork pâtés sampled in Canada or 4% of pork livers bought in butcher shops and supermarkets in southern Germany [18,22,23]. Cell culture models, which could distinguish between non-infectious RNA-fragments and infective virus, are not yet generally available. Thus, epidemiological findings are needed to help assess these products actual risk to the consumer. Our results indicate that HEV is insufficiently inactivated during common sausage production methods. A study applying a bioassay showed that HEV is resistant to heat and recommended that pork, pork products as well as meat products from wild animals such as boar be heated to ≥ 71 °C for a duration of at least 20 min to inactivate HEV [17].

Ready-to-eat pork products have also been found to be a risk factor in England, where the consumption of pork pie, sausages and ham were significantly associated with infection [4]. Pork liver pâtés have been implicated in an outbreak of hepatitis E in Australia [24]. Food habits, preferences and available food items vary enormously even between European countries and more research is needed to determine risk factors common and unique to different countries and populations.

The association between the consumption of raw vegetables and disease in our study was unexpected. In the EU and most other countries, the use of pig manure is closely regulated to avoid harm to the consumer. Moreover, lettuce, which is grown, marketed and consumed similarly to raw vegetables, was not associated with disease in our study. We thus regard this finding as possibly accidental and warranting further research.

The significant difference in the proportion of persons avoiding pork or pork products for religious or other reasons among cases (0.4%) and matched controls (5.8%) and the striking population attributable fraction of 93.9% lends another line of evidence for the consumption of pork and pork products as the major risk factor for acquiring zoonotic (genotype 3) hepatitis E in Germany. It also argues against a high contribution of vegetarian food items to the burden of infections.

In addition to risk factors related to exposure to HEV, several pre-existing conditions were strongly associated with hepatitis E. These included pre-existing liver disease which might predispose hepatitis E patients to severe courses of disease [12,25,26], diabetes mellitus which likely impairs hepatocyte-regenerating capacity [27] and (non-inflammatory) gastrointestinal diseases, which have, not previously been reported as associated with hepatitis E. The nature of the association of the latter is unknown but could be related to intake of acid-reducing drugs (e.g. proton pump inhibitors) which was reported by more than half of the patients with pre-existing non-inflammatory conditions of the gastrointestinal system in our study.

### Clinical aspects

Our data shows that acute hepatitis E is the source of considerable morbidity and cost, with 73.7% of case-patients hospitalised and a median of 20 days of missed work among those employed. This is considerably more than in German hepatitis A patients interviewed in the framework of an enhanced surveillance study, where 46% were hospitalised and those employed missed a median of 6 days of work [28]. The incidence of hepatitis E in our study was more than twice as high as the incidence of hepatitis A in Germany in 2016 [14].

While there is no sex difference in terms of HEV seroprevalence in Germany [15], 60% of cases notified between 2012 and 2016 were diagnosed in men [14]. It is unclear whether this is due to sex-specific susceptibility [29], differences in the prevalence of additional risk factors for developing clinical hepatitis (i.e. pre-existing liver conditions) [25], differential diagnostic work-up of disease in men and women or whether men are exposed to higher alimentary doses of HEV compared with women and are thus more likely to develop symptoms. Our data show statistically significant differences in the clinical presentation and hospitalisation proportion of acute hepatitis E in men and women, suggesting that there are indeed sex-specific differences in disease development.

Diagnosing hepatitis E in a patient should be straightforward. There are several serological and PCR-based assays on the market and available to health practitioners in Germany through professional laboratories [30]. Our data shows that physicians usually suspected (viral) hepatitis and took a blood sample at the patient’s first visit. However, there were often long delays before the diagnosis of hepatitis E was made. It seems that hepatitis E was not considered as a relevant differential diagnosis or was only considered after other causes were ruled out. Hepatitis E has long been regarded as a rare, imported disease [31] and in this situation it appeared to be economical to initially test for the more common hepatitis A, B and C virus, cytomegalovirus and Epstein–Barr virus infection in patients with hepatitis or elevated liver enzymes. Today, hepatitis E may be the most frequently diagnosed viral cause of hepatitis in patients seen by medical specialists and general practitioners [32] and many laboratories have consequently included HEV in their routine hepatitis work flow. Still, recent data shows that patients with acute hepatitis caused by HEV are frequently misdiagnosed as having drug-induced liver injury or auto-immune hepatitis [33,34]. The high proportion of patients in our study who received invasive diagnostic procedures (gastro- or colonoscopy: 19%; biopsy of the liver: 17%) or visited more than one physician before hepatitis E was diagnosed, suggests that practitioners failed to consider HEV infection as a differential diagnosis right away.

### Limitations

Due to the high lifetime prevalence of symptomatic or asymptomatic HEV infection in Germany [15], we have to assume that several control persons were seropositive at the time of the study and thus were not susceptible to HEV exposure in the 2 months before the interview. This likely leads to an underestimation of odds ratios (ORs) of relevant risk factors or that a risk factor remains undetected. ORs reported in our study should thus be seen as a conservative estimate of the true ORs (or risk).

An important potential source of bias in all case–control studies is differential recall between cases and controls. While cases were interviewed about exposures 2 months before symptom onset, we chose the 2 months before the interviews for controls to adjust for the more vivid memory of the period before disease onset in cases.

## Conclusions and recommendations

Our population-based study suggests that the consumption of pork and pork products is the main risk factor for symptomatic hepatitis E infections acquired in Germany. This specifically includes products that are intended to be consumed without further preparation by the consumer (such as liver sausage). The study expands on the role of host factors that play a considerable role in determining who develops clinical hepatitis E and who remains asymptomatic after an exposure to HEV, such as pre-existing liver disease or diabetes mellitus.

We recommend educating consumers that pork and offal should always be thoroughly cooked before consumption. Further epidemiological and virological studies are needed to confirm the role of cooked or parboiled sausages and raw vegetables as a possible risk factor for hepatitis E and to investigate sources of contamination and methods of virus detection and inactivation. In view of accumulating evidence of commercial pork products as one of the main risk factors for hepatitis E, production methods should be reviewed and possibly adjusted to ensure inactivation of HEV. Opportunities to reduce HEV in commercial pig herds should be explored. Physicians need to be aware that testing for hepatitis E is indicated in all cases of clinical hepatitis in order to prevent delays in diagnosis.
